# Wolfberry genome database: integrated genomic datasets for studying molecular biology

**DOI:** 10.3389/fpls.2024.1310346

**Published:** 2024-02-20

**Authors:** You-Long Cao, You-Yi Chen, Yan-Long Li, Chung-I Li, Shao-Ting Lin, Bing-Ru Lee, Chun-Lin Hsieh, Yu-Yun Hsiao, Yun-Fang Fan, Qing Luo, Jian-Hua Zhao, Yue Yin, Wei An, Zhi-Gang Shi, Chi-Nga Chow, Wen-Chi Chang, Chun-Lin Huang, Wei-Hung Chang, Zhong-Jian Liu, Wei-Sheng Wu, Wen-Chieh Tsai

**Affiliations:** ^1^ National Wolfberry Engineering Research Center, Ningxia Academy of Agriculture and Forestry Sciences, Yinchuan, China; ^2^ Institute of Wolfberry Engineering Technology, Ningxia Academy of Agriculture and Forestry Sciences, Yinchuan, China; ^3^ Department of Agronomy, National Chiayi University, Chiaiyi, Taiwan; ^4^ Department of Statistics, National Cheng Kung University, Tainan, Taiwan; ^5^ Graduate Program in Translational Agricultural Sciences, National Cheng Kung University and Academia Sinica, Tainan, Taiwan; ^6^ Department of Electrical Engineering, National Cheng Kung University, Tainan, Taiwan; ^7^ Orchid Research and Development Center, National Cheng Kung University, Tainan, Taiwan; ^8^ Institute of Tropical Plant Sciences and Microbiology, National Cheng Kung University, Tainan, Taiwan; ^9^ Department of Biology, National Museum of Natural Science, Taichung, Taiwan; ^10^ Department of Psychiatry, National Cheng Kung University Hospital, Collage of Medicine, National Cheng Kung University, Tainan, Taiwan; ^11^ Department of Psychiatry, National Cheng Kung University Hospital, Douliu, Taiwan; ^12^ Key Lab of National Forestry and Grassland Administration for Orchid Conservation and Utilization and International Orchid Research Center at College of Landscape Architecture, Fujian Agriculture and Forestry University, Fuzhou, Fujian, China; ^13^ Zhejiang Institute of Subtropical Crops, Zhejiang Academy of Agricultural Sciences, Wenzhou, China; ^14^ Institute of Vegetable and Flowers, Shandong Academy of Agricultural Sciences, Jinan, China; ^15^ Department of Life Sciences, National Cheng Kung University, Tainan, Taiwan; ^16^ University Center for Bioscience and Biotechnology, National Cheng Kung University, Tainan, Taiwan

**Keywords:** wolfberry, *Lycium barbarum*, wolfberry genome database (WGDB), user-friendly, genomics, transcriptomics

## Abstract

Wolfberry, also known as goji berry or *Lycium barbarum*, is a highly valued fruit with significant health benefits and nutritional value. For more efficient and comprehensive usage of published *L. barbarum* genomic data, we established the Wolfberry database. The utility of the Wolfberry Genome Database (WGDB) is highlighted through the Genome browser, which enables the user to explore the *L. barbarum* genome, browse specific chromosomes, and access gene sequences. Gene annotation features provide comprehensive information about gene functions, locations, expression profiles, pathway involvement, protein domains, and regulatory transcription factors. The transcriptome feature allows the user to explore gene expression patterns using transcripts per kilobase million (TPM) and fragments per kilobase per million mapped reads (FPKM) metrics. The Metabolism pathway page provides insights into metabolic pathways and the involvement of the selected genes. In addition to the database content, we also introduce six analysis tools developed for the WGDB. These tools offer functionalities for gene function prediction, nucleotide and amino acid BLAST analysis, protein domain analysis, GO annotation, and gene expression pattern analysis. The WGDB is freely accessible at https://cosbi7.ee.ncku.edu.tw/Wolfberry/. Overall, WGDB serves as a valuable resource for researchers interested in the genomics and transcriptomics of *L. barbarum*. Its user-friendly web interface and comprehensive data facilitate the exploration of gene functions, regulatory mechanisms, and metabolic pathways, ultimately contributing to a deeper understanding of wolfberry and its potential applications in agronomy and nutrition.

## Background

Wolfberry, also known as goji berry or *Lycium barbarum*, is a bright red or orange–red berry that belongs to the *Lycium* genus. The *Lycium* genus comprises approximately 100 species that are distributed from temperate to subtropical regions. These plants have various adaptations, such as drought, salt, and temperature tolerance, which allow them to thrive in diverse environments (Yao et al., 2018). Wolfberry has been used in traditional Chinese medicine for centuries due to its numerous health benefits. In recent years, it has gained popularity worldwide as a superfood because of its rich nutritional profile and potential health-promoting properties. Wolfberries are packed with essential nutrients and bioactive compounds, making them a valuable addition to a balanced diet. They are a rich source of vitamins, minerals, antioxidants, and phytochemicals (Vidović et al., 2022). Some of the prominent nutrients found in wolfberries include vitamin C, vitamin A, iron, zinc, fibre, zeaxanthin, and polysaccharides (Vidović et al., 2022).

While many researchers have focused on the functional ingredients and potential industrial applications of wolfberry, there has been increasing attention given to genetic and genomic approaches in understanding the molecular mechanisms of fruit development, nutrient accumulation, biosynthesis of bioactive compounds, physiological processes, and stress tolerance in wolfberry. For example, the transcriptional ratio of branch node structural genes F3’5’H/F3’H may determine the phenotypic difference in anthocyanin biosynthesis between *L. ruthenicum* and *L. barbarum* fruits (Zhen et al., 2014). Additionally, a study identified 137 R2R3-MYB genes in the wolfberry genome, and Lba11g0183 and Lba02g01219 were suggested as candidate R2R3-MYB genes regulating carotenoid biosynthesis in wolfberry (Yin et al., 2022). Another study compared the transcriptome, metabolome, and hormone changes between *L. chinense* and *L. ruthenicum* and found that ABA (abscisic acid) and flavones might play positive roles in resistance to salinity stress in wolfberry (Qin et al., 2022).

In 2021, the genome of wolfberry *L. barbarum* was sequenced using a whole-genome shotgun strategy and PacBio technology. This species has 24 chromosomes, and the assembled genome size is 1.67 Gb, with a contig N50 value of 10.75 Mb. High-throughput/resolution chromosome conformation capture (Hi-C) technology was adopted to obtain the lengths of the 12 chromosomes, which ranged from 106.53 to 172.84 Mb. In total, 33,581 genes and 151 miRNAs were annotated in the *L. barbarum* genome (Cao et al., 2021). Molecular biologists or agronomists who are interested in the wolfberry genome could access the genome sequences from the NCBI database. However, many of them might not have appropriate knowledge or analysis skills to explore the biological significance embedded in the genetic sequences. The WGDB encompasses not only the genome and transcriptome information of *L. barbarum* but also the annotated gene position at the specific location of the genome, functional annotation in various databases (structure database, GO, and KEGG database), transcriptional regulation (promoter and transcription factor), and posttranscriptional regulation (miRNA and target mRNA). Based on this dataset, a wolfberry *L. barbarum* database was established in this study to provide easy-to-use web interfaces for accessing this comprehensive information.

## Methods

### Construction and content

The architecture of the WGDB is designed to store and analyze *L. barbarum* genome sequences and annotation data. It consists of several components: 1. MySQL 5.7 Database: This is used to store the collected *L. barbarum* genome sequences and their corresponding annotation data. MySQL is a popular open-source relational database management system. 2. Django 1.11.1 Application: Django is a Python web framework that handles the execution of all the analysis tasks in the WGDB. It provides a high-level abstraction for building web applications. 3. Web Interface: The web interface of the WGDB is constructed using HTML and JavaScript. It allows the user to interact with the database, access and visualize the stored data, and perform various analysis tasks. 4. DataTables: WGDB utilizes the DataTables plugin in the jQuery JavaScript library to generate tables for displaying and interacting with data in the web interface. DataTables provide features such as sorting, searching, and pagination for efficient data presentation. 5. D3.js: To generate interactive plots of synteny and gene order, WGDB employs D3.js, a powerful JavaScript library for creating dynamic and data-driven visualizations. D3.js enables the creation of custom visualizations based on the retrieved data.

**Figure 1:**
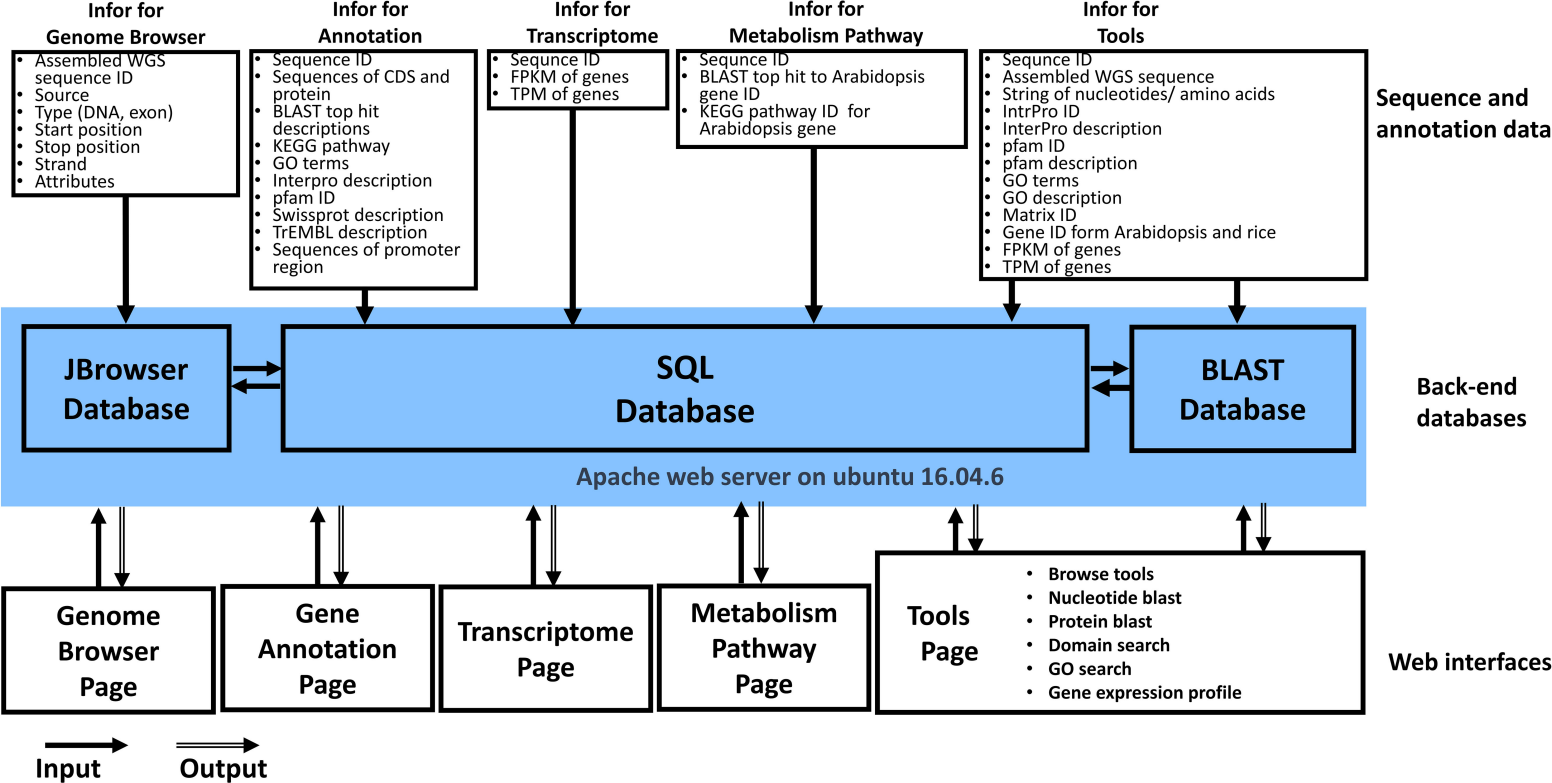
Overview of Wolfberry Genome Database architecture.

The WGDB is deployed on a workstation running the Linux operating system, specifically Ubuntu 16.04.6. The hardware configuration of the workstation includes two CPUs, 55 GB RAM, and 3.6 TB of hard disk space. This setup allows for efficient storage, processing, and analysis of large and complex biological datasets. **Figure 1** shows an overview of the database architecture. In addition to the mentioned components, the WGDB integrates various data and tools summarized in **Table 1**. These resources include genome browsers such as JBrowse for navigating and visualizing *L. barbarum* genomes, as well as additional databases such as BLAST for sequence similarity searches. Overall, the WGDB serves as an open-access and web-available portal that simplifies the workflow for analyzing and visualizing the genomes of *L. barbarum* and related transcriptome information.

**Table 1:** Summary of data and tools that could be browsed and used for the Wolfberry (Lycium barbarum).

Genome browser	Scaffold ID, Scaffold sequence, Gene model
Gene annotation	Gene ID, Gene sequence, BLAST top hit descriptions, KEGG pathway, GO terms, Interpro description, Pfam description, Swissprot description, TrEMBL description, Number of predicted target genes from Arabidopsis and rice
Transcriptome	Gene ID, FPKM and TPM values in various tissues
Metabolism pathway	Gene ID, Genes mapped to the KEGG pathways
Tools	
Browse tools	IntrPro ID, InterPro description, Pfam ID, Pfam description, GO terms, GO description, Matrix ID, Gene ID from Arabidopsis and rice, Number of predicted target genes
Nucleotide blast	BLASTN, tBLASTX, tBLASTN
Protein blast	BLASTX, BLASTP
Domain search	Pfam ID, Pfam description, Searching for related genes ID
GO search	GO terms, GO description, Searching for related genes ID
Gene expression profile	Gene ID, FPKM and TPM values in various tissues, Heat map, expression pattern, PCA, Cluster

### The database content

*L. barbarum* has a karyotype of 2N = 2X = 24 chromosomes. The assembled genome total length is 1.67 Gb with an N50 value of 10.75 Mb, which encompasses 12 pseudomolecules (Cao et al., 2021). We also evaluated assembly continuity of the *L. barbarum* genome by PlantLAI (https://bioinformatics.um6p.ma/PlantLAI), and got the raw LAI score 12.67 and the LAI value 12.22 of the assembled genome. This results indicated the *L. barbarum* genome is a reference quality genome (Ou et al., 2018; Mokhtar et al., 2023). The 33,581 protein-coding genes were predicted and assigned a specific Gene ID to serve as a unique identifier. These Gene IDs enable the investigation of annotated functions of individual genes. The WGDB allows the user to select specific genes and explore their annotated functions, particularly those related to biological processes of interest.

The database also includes transcriptomics data obtained from RNA sequencing (RNA-seq) experiments performed on *L. barbarum*. The RNA-seq data were derived from various developmental stages of flowers and fruits, mature leaves, flowers, stems, and roots. The RNA-seq data that had been sequenced in triplicate for each collected sample were downloaded from PRJNA788208 at NCBI. Clean reads of each sample were assessed by FastQC (version 0.12.1). The R package Rsubread (version 2.16.0) was used to map the *clean* reads in the fastq files to the reference genome and calculate the raw read count matrix. The clean read counts were then mapped to the predicted genes, and transcripts per million (TPM) and fragments per kilobase of transcript per million mapped reads (FPKM) values were calculated. These TPM and FPKM values represent the gene expression levels in different tissues and developmental stages. The integration of gene expression data into the WGDB enables the user to analyze and explore gene expression patterns in *L. barbarum*.

Overall, the WGDB provides access to a wide range of data, including the *L. barbarum* genome assembly, predicted genes and miRNAs, gene annotations, and gene expression information derived from RNA-seq experiments. This comprehensive collection of data facilitates the study of *L. barbarum* biology and the exploration of gene functions and regulatory mechanisms in this species.

### Utility and discussion

**Figure 2:**
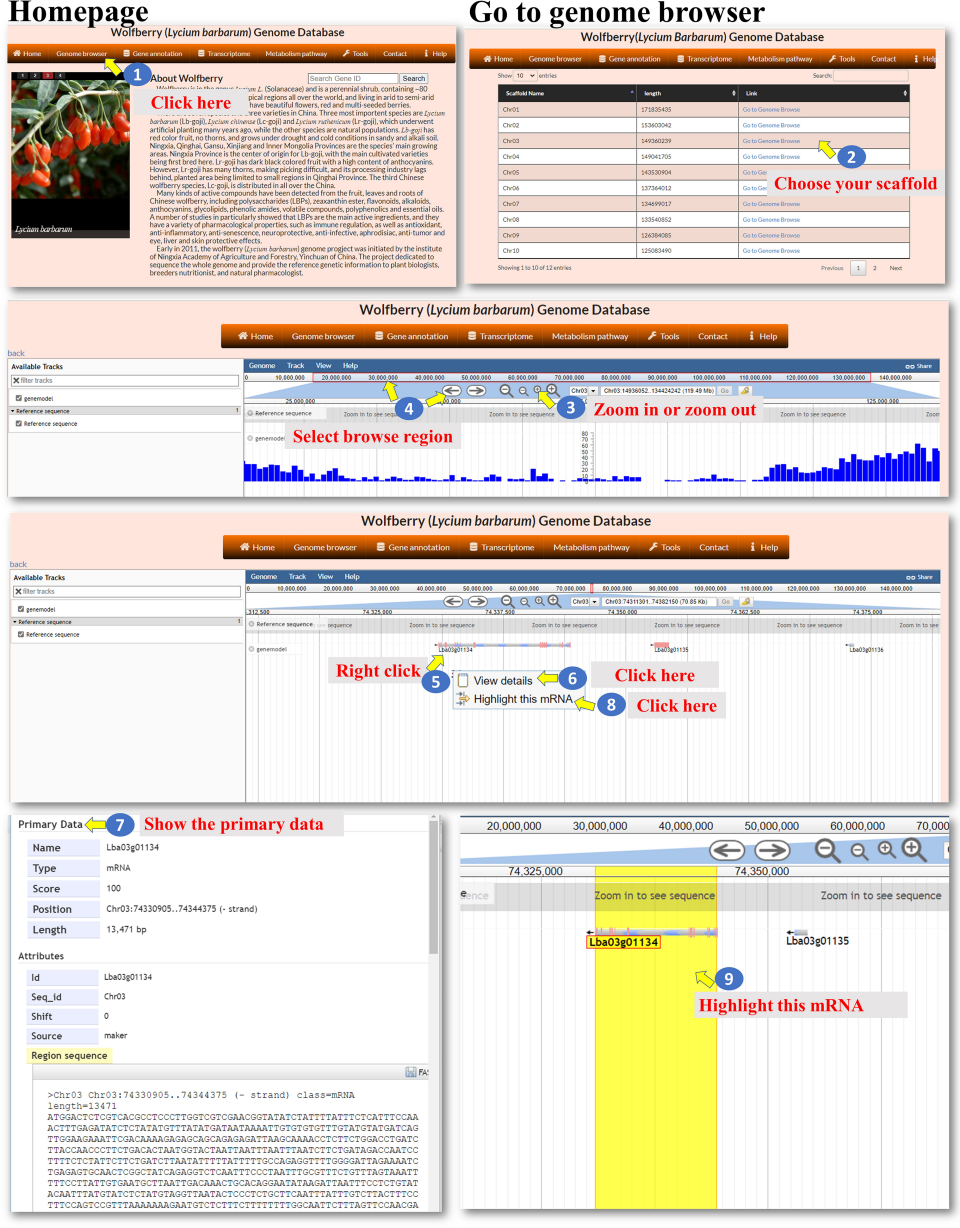
Step-by-step guide for the Genome browser page.

The WGDB is a highly efficient, web-accessible relational database with comprehensive genomic information. Through the web interface, the genome information of *L. barbarum* in the database could be freely obtained. The information can be linked via the “https://cosbi7.ee.ncku.edu.tw/Wolfberry/” website (**Figure 2**).

**Genome Browser**: The Genome browser interface allows the user to explore the *L. barbarum* genome and browse the sequences of specific chromosomes. In the first step, the user enters the *L. barbarum* genome into the Genome browser interface (**Figure 2**, step 1). The user can select one of the chromosomes they want to explore. This selection allows them to focus on a specific chromosome’s sequences (**Figure 2**, step 2). After selecting a chromosome, the user can zoom in or zoom out on the genome sequence (**Figure 2**, step 3). Zooming in allows for a closer view of the DNA sequence, while zooming out provides a broader overview. To navigate through the genome, the user can utilize the “arrowhead” feature. Clicking on the arrowhead allows the user to move along the genome sequence, either forwards or backwards, to browse different positions (**Figure 2**, step 4). While browsing the genome, the user can identify regions containing genes. They can move the cursor to a specific gene of interest and perform various actions (**Figure 2**, step 5). By clicking the right button of the mouse, the user can retrieve the gene sequence associated with the selected gene. This action might display the nucleotide sequence or provide a downloadable file containing the gene’s DNA sequence (**Figure 2**, steps 6 to 7). Alternatively, the user may choose to highlight the gene of interest. This action visually distinguishes the gene from the surrounding sequence, making it easier to identify and analyze (**Figure 2**, steps 8 to 9). By combining these features, the user can navigate the *L. barbarum* genome, explore specific chromosomes, zoom in or out, browse positions, and access gene sequences or highlight genes for further analysis.

**Figure 3:**
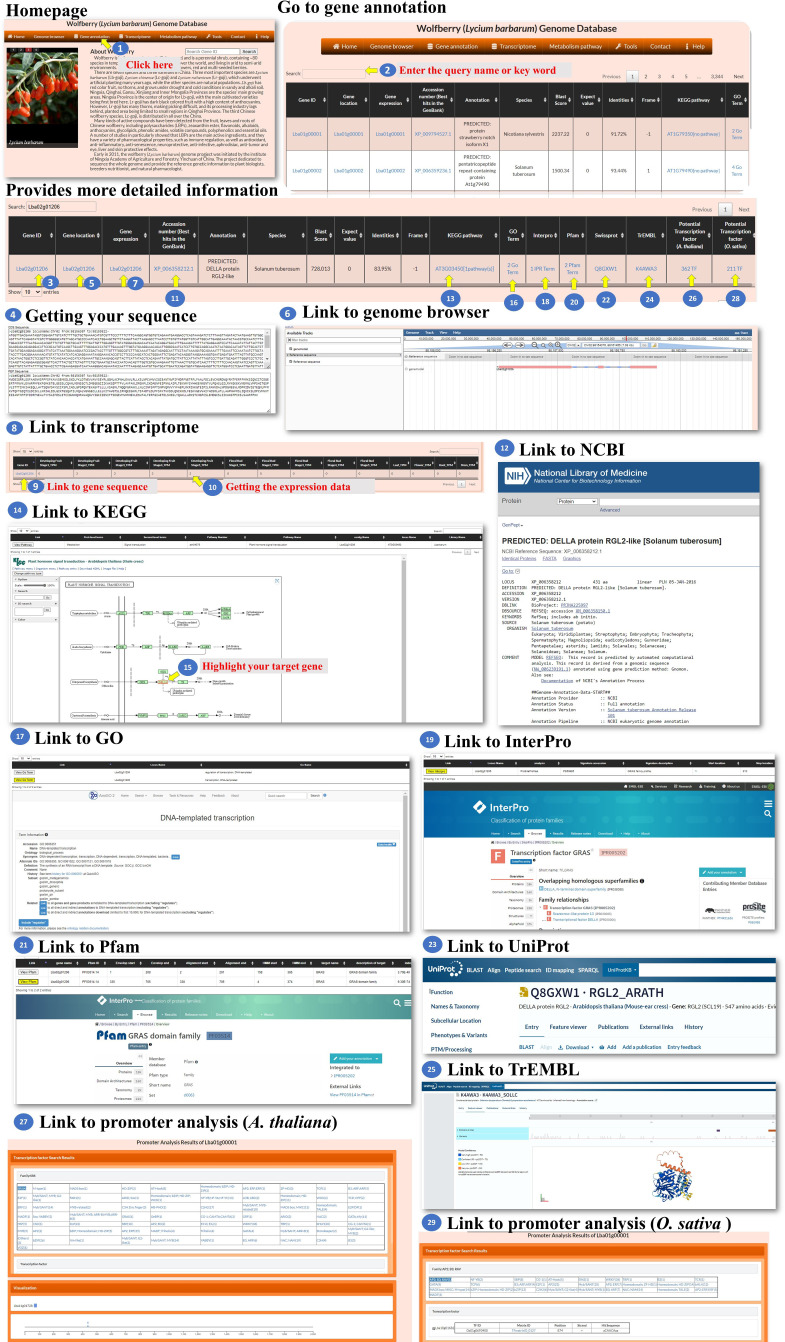
Step-by-step guide for the Gene annotation page.

**Gene annotation**: The gene annotation process involves accessing various pieces of information about genes within *L. barbarum*. The BLASTP programs were used to select the best hit in the NR or TrEMBL database with the smallest E-value, and the E-value must be less than 10^-5^. The user could access the information of the genes in this page (**Figure 3**, step 1). To retrieve information about a specific gene, the user can enter the Gene ID into a search box (**Figure 3**, step 2). This ID serves as a unique identifier for the gene of interest. Upon querying the Gene ID, the user can access the gene sequence linked to that particular ID (**Figure 3**, steps 3-4). This sequence could be displayed or made available for download. To determine the specific location of the gene on the chromosome, the user can retrieve the gene’s location information (**Figure 3**, steps 5-6). These data provide the chromosome number and the position of the gene within that chromosome. The user can access the expression profile of the gene (**Figure 3**, steps 7-10). The user can retrieve the best BLAST hit in the NCBI database, which refers to the gene’s closest matching sequence in the NCBI database (**Figure 3**, steps 11-12). The accession number allows the user to access additional information about this hit. The user can explore the gene’s involvement in a biological pathway using the KEGG pathway database (**Figure 3**, steps 13-15). This information helps understand the role of genes within a specific biological context. The gene’s function can be classified using gene ontology (GO) terms (**Figure 3**, steps 16-17). GO terms provide standardized annotations that describe the gene’s functional attributes. The KEGG and GO pathways of a wolfberry gene are annotated using the KEGG and GO pathways of its orthologues in *Arabidopsis*. The orthologues in *Arabidopsis* for a wolfberry gene are assigned by the best hit of running BLASTP against all *Arabidopsis* protein sequences. The best hit is defined as the one with the smallest E-value, and the E-value must be less than 10^-5^. The user can access information about functional protein domains associated with the gene using resources such as Interpro, Pfam, or Swisspro (**Figure 3**, steps 18-23). These domains provide insights into protein structure and function. The Pfam/InterPro domains of a wolfberry protein were identified using HMMER/InterProScan tools (Quevillon et al., 2005). Similar to the NCBI database, the user can retrieve the best BLAST hit in the EMBL-EBI database (**Figure 3**, steps 24-25). This hit provides additional information and context for the gene of interest. The user can explore the gene’s regulation by transcription factors and their binding sites within the promoter. This information may be available for both Arabidopsis and rice orthologues (**Figure 3**, steps 26-29). This study provides insights into the regulatory mechanisms controlling gene expression. The promoter of a wolfberry gene is defined as the 2,000 bp upstream of the start codon of a gene. The binding sites of all *Arabidopsis*/rice (transcription factors) TFs in a given promoter were identified using the promoter analysis tool in PlantPAN 3.0 (Chow et al., 2019). After running the promoter analysis tool, all the *Arabidopsis*/rice TFs whose binding sites at the promoter of a wolfberry gene of interest could be screened. Then, the best hits of *L. barbarum* TFs in *Arabidopsis*/rice were identified by BLASTP. Finally, we recommended that these Wolfberry TFs might bind to the promoter of a Wolfberry gene of interest. By following these steps, the user can gather comprehensive information about genes, including their sequences, locations, expression profiles, database hits, pathway involvement, functional classifications, protein domains, regulatory factors and binding sites.

**Figure 4:**
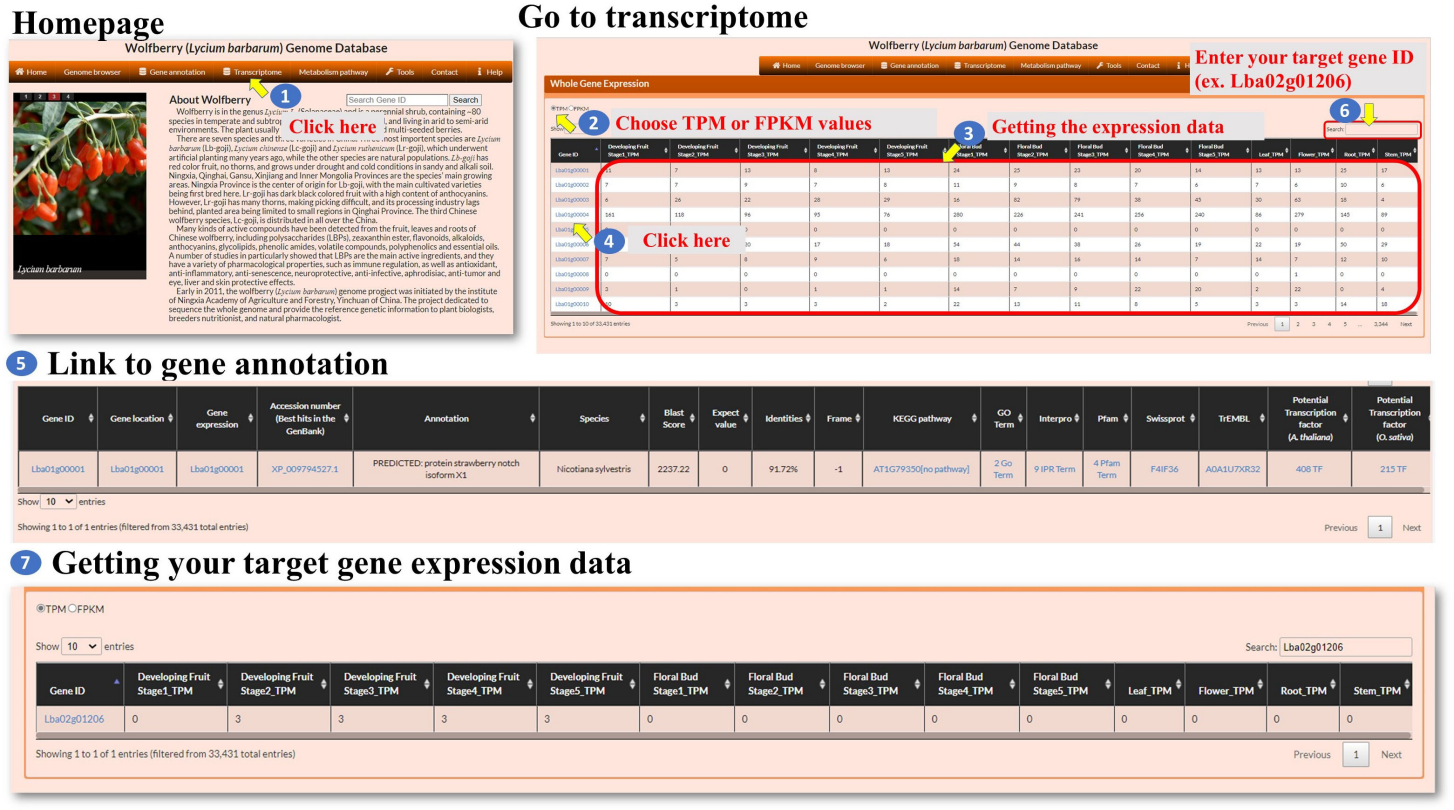
Step-by-step guide for the Transcriptome page.

**Transcriptome:** The transcriptome feature within the interface allows the user to explore the expression patterns of genes. The user can click on the “Transcriptome” option (**Figure 4**, step 1) to navigate to the Transcriptome page. This page provides information about gene expression. On the Transcriptome page, the user can select the expression level metric they want to use, either TPM (transcripts per million) or FPKM (fragments per kilobase of transcript per million mapped reads) (**Figure 3**, steps 2-3). These metrics are commonly used normalization methods in RNA sequencing (RNA-seq) data analysis. It helps to account for differences in sequencing depth and gene length across samples. TPM values represent the relative abundance of a transcript in a sample (Zhao et al., 2020). FPKM is similar to TPM but uses the concept of fragments instead of transcripts (Zhao et al., 2021). The Gene ID associated with each gene can be linked to its annotation (**Figure 3**, steps 4-5). This allows the user to access additional information about the selected genes, such as gene function, biological pathways, or protein domains. The Transcriptome page provides a search function at the top right of the page (**Figure 3**, steps 6-7). The user can enter a specific Gene ID into the search box to retrieve the gene expression patterns for the searched gene. This allows the user to quickly find and analyze the expression of a specific gene of interest. Overall, the transcriptome feature provides the user with the ability to explore gene expression patterns. The user can select between TPM and FPKM as expression level metrics, link Gene IDs to gene annotations, and utilize the search function to retrieve expression patterns for specific genes.

**Figure 5:**
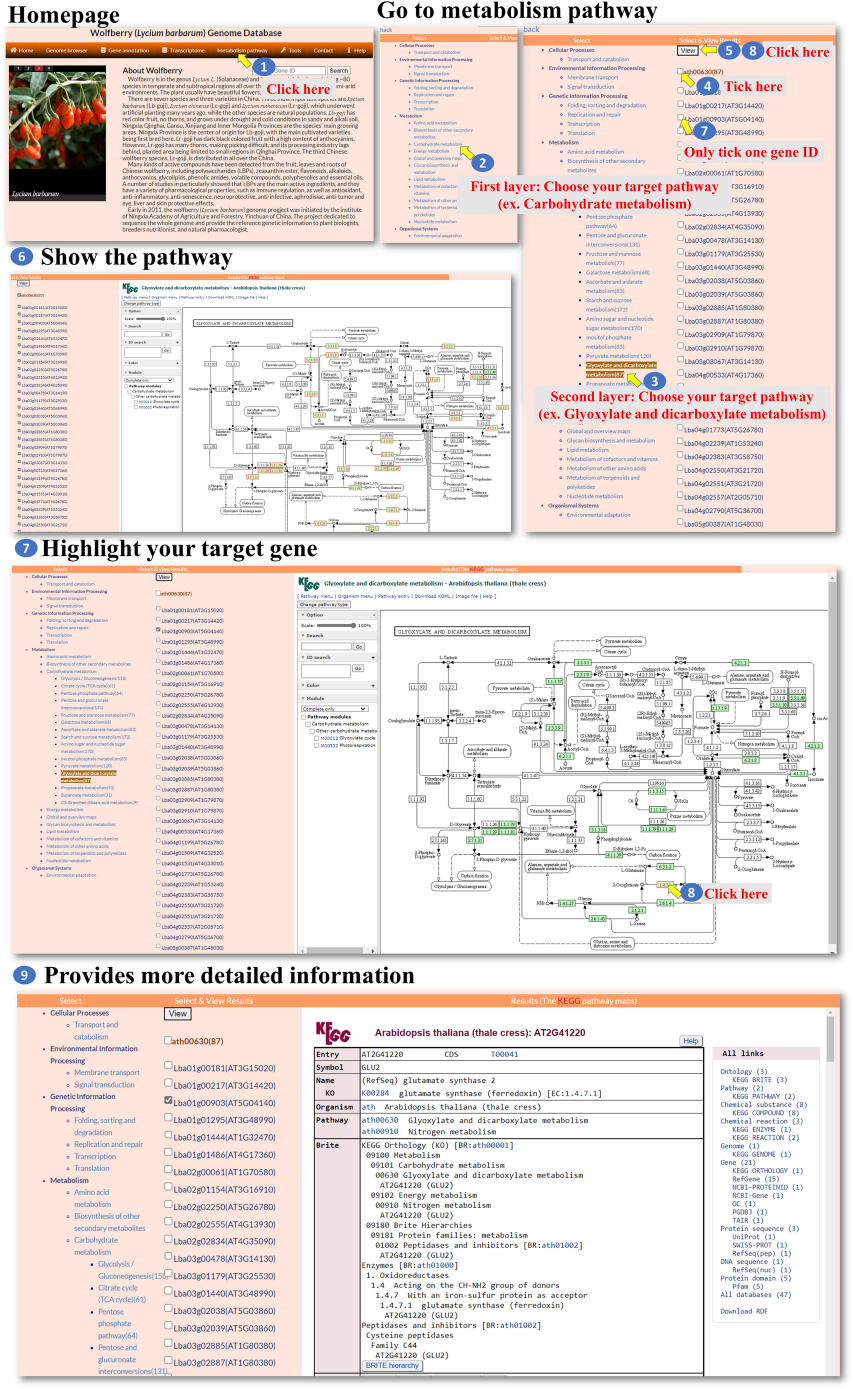
Step-by-step guide for the Metabolism pathway page.

**Metabolism pathway:** The “Metabolism pathway” page within the interface provides information about selected genes involved in KEGG pathways. The user can navigate to the “Metabolism pathway” page to explore metabolic pathways and their associated genes (**Figure 5**, step 1). On the “Metabolism pathway” page, the user can select one of the major pathways and then choose a specific subpathway of interest (**Figure 5**, steps 2-3). These pathways represent different metabolic processes or branches within metabolism. Once a pathway and subpathway are selected, the user can choose one or multiple Gene IDs associated with genes in that pathway. Clicking the “View” icon (**Figure 5**, steps 4-8) allows the user to generate an image that shows the selected pathway, with enzymes involved in the pathway marked in red. This image is derived from the KEGG database. The generated colored pathway image is interactive (**Figure 5**, step 9). The user can interact with the image to access the KEGG database and explore additional information about the enzymes, pathways, and related metabolic processes. This feature allows the user to delve deeper into the specific metabolic pathways and their associated genes. Overall, the “Metabolism pathway” page enables the user to explore metabolic pathways and the involvement of selected genes within those pathways. The user can select major pathways and subpathways, view pathway images with marked enzymes, and access the KEGG database for further exploration and information retrieval.

**Figure 6:**
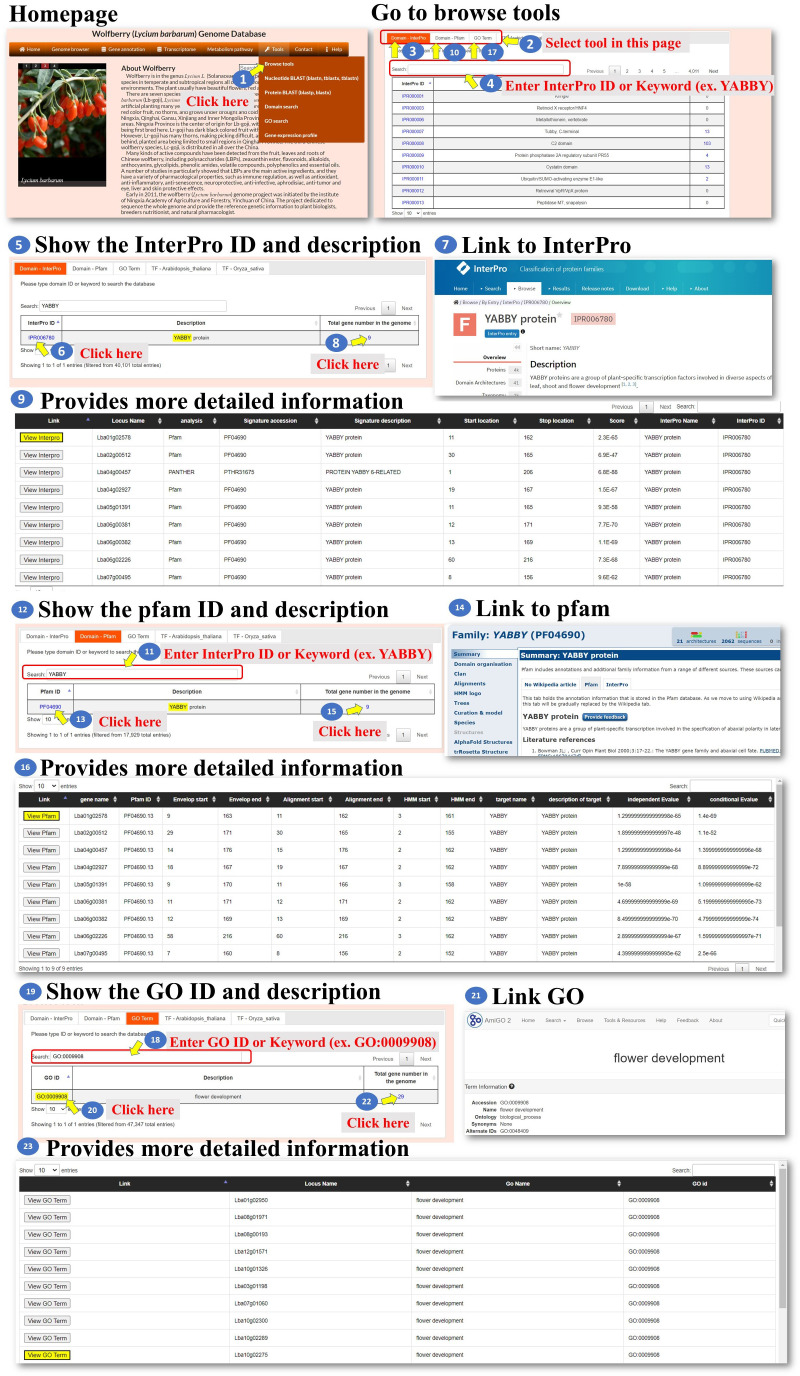
Step-by-step guide for the Tools Browse tools including Domain_Interpro, Domain_Pfam, and Domain_GO.

**Tools:** We developed six tools for the analysis of the *L. barbarum* genome and gene function. These tools encompass various functionalities to assist the user in their analysis (**Figure 6** step 1). Under the “Browse tools” section, there are five subtools available. These subtools offer different approaches to analyze the genome and gene function of *L. barbarum* (**Figure 6**, step 2). In the case of the “Domain-InterPro” tool, the user can either browse each page or input an InterPro domain ID or InterPro domain keyword. This tool allows the user to search for specific InterPro domains related to their analysis. As an example, the keyword “YABBY” is input (**Figure 6**, steps 3 to 5). When the user clicks on the InterPro ID (blue color), they will be directed to the InterPro database, where they can access detailed information about the InterPro domain they selected. This step helps the user obtain comprehensive information about the domain they are interested in (**Figure 6**, steps 6 to 7). Additionally, the page provides a whole genome analysis of the genes encoding the indicated domain. This feature allows the user to explore and analyze the genes associated with the selected InterPro domain across the entire *L. barbarum* genome (**Figure 6**, steps 8 to 9). Finally, the steps involved in using the “Domain-Pfam” and “GO Term” tools are similar to the process described for the “Domain-InterPro” tool. The user can follow the instructions provided, likely involving searching for specific domains or terms, accessing detailed information, and conducting genome-wide analyses (**Figure 6**, steps 10 to 22). Overall, these tools offer a range of functionalities for analyzing the *L. barbarum* genome and gene function, with specific features for searching, obtaining domain details, and conducting genome-wide analyses related to InterPro domains, Pfam domains, and GO terms.

**Figure 7:**
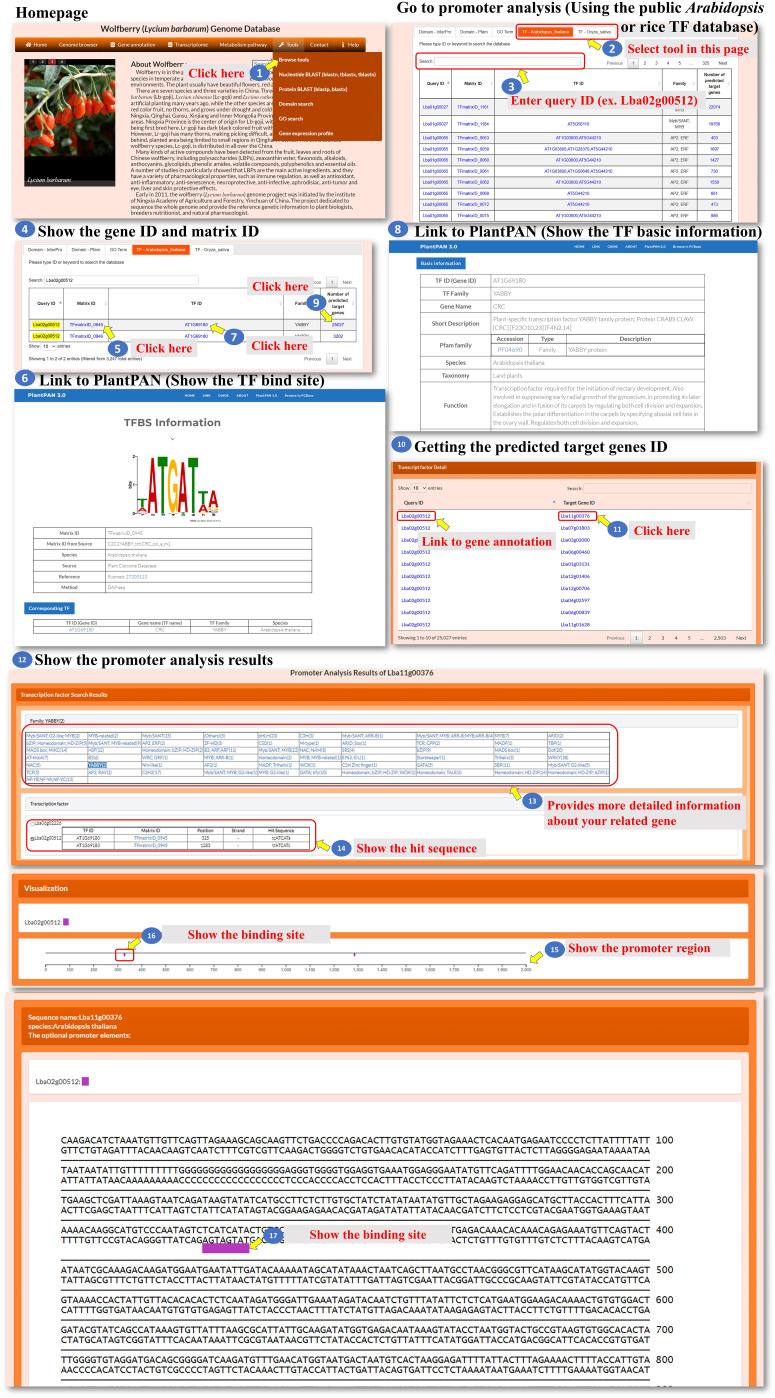
Step-by-step guide for the Tools_Browse tools including TF-Arabidopsis thaliana and TF-Oryza sativa.

All the genes encoding transcription factors have been identified, and their binding sites in the *L. barbarum* genome and orthologues in *Arabidopsis* and rice were also predicted as well as their putative target genes. The user can click on either “TF-*Arabidopsis thaliana*” or “TF-*Oryza sativa*” to access the respective pages. These pages contain information about the identified transcription factors and their characteristics (**Figure 7**, steps 1 to 4). The predicted binding sites of *L. barbarum* transcription factors are linked to PlantPAN 3.0 (Chow et al., 2019). Clicking on the binding site redirects the user to the PlantPAN 3.0 database, where they can access additional information and analysis related to the predicted binding sites (**Figure 7**, steps 5 to 6). The orthologues of *Arabidopsis* or rice for the identified *L. barbarum* transcription factors are indicated (**Figure 7**, steps 7 to 8). This information helps the user understand the relationships and similarities between the transcription factors in different plant species. The table includes a column labelled “Number of predicted target genes”. This column provides the count of predicted downstream genes that are regulated by the transcription factor encoded by the *L. barbarum* gene. The user can click on the number in the column to proceed to further analysis (**Figure 7**, steps 9 to 11). Clicking on the specific number of predicted target genes navigates the user to a table that presents the predicted target gene IDs (**Figure 7**, step 11). This table helps the user identify and explore the specific genes regulated by the transcription factor of interest. By clicking on a specific target gene ID, the user can access information about the transcription factor genes that regulate the target gene (**Figure 7**, step 12). The information provided for each target gene includes the binding sequence of the transcription factor that regulates it (**Figure 7**, step 13). Additionally, the position of the binding sequence within 2,000 bp upstream of the target gene promoter is indicated (**Figure 7**, steps 14 to 17). These details help the user understand the regulatory mechanisms involved in the interaction between transcription factors and their target genes. In summary, the tools we have developed allow the user to explore and analyze the transcription factors in *L. barbarum*, including their predicted binding sites, orthologues in *Arabidopsis* and rice, and putative target genes. The provided functionalities enable the user to delve into the regulatory relationships between transcription factors and their targets in a convenient and effective manner.

**Figure 8:**
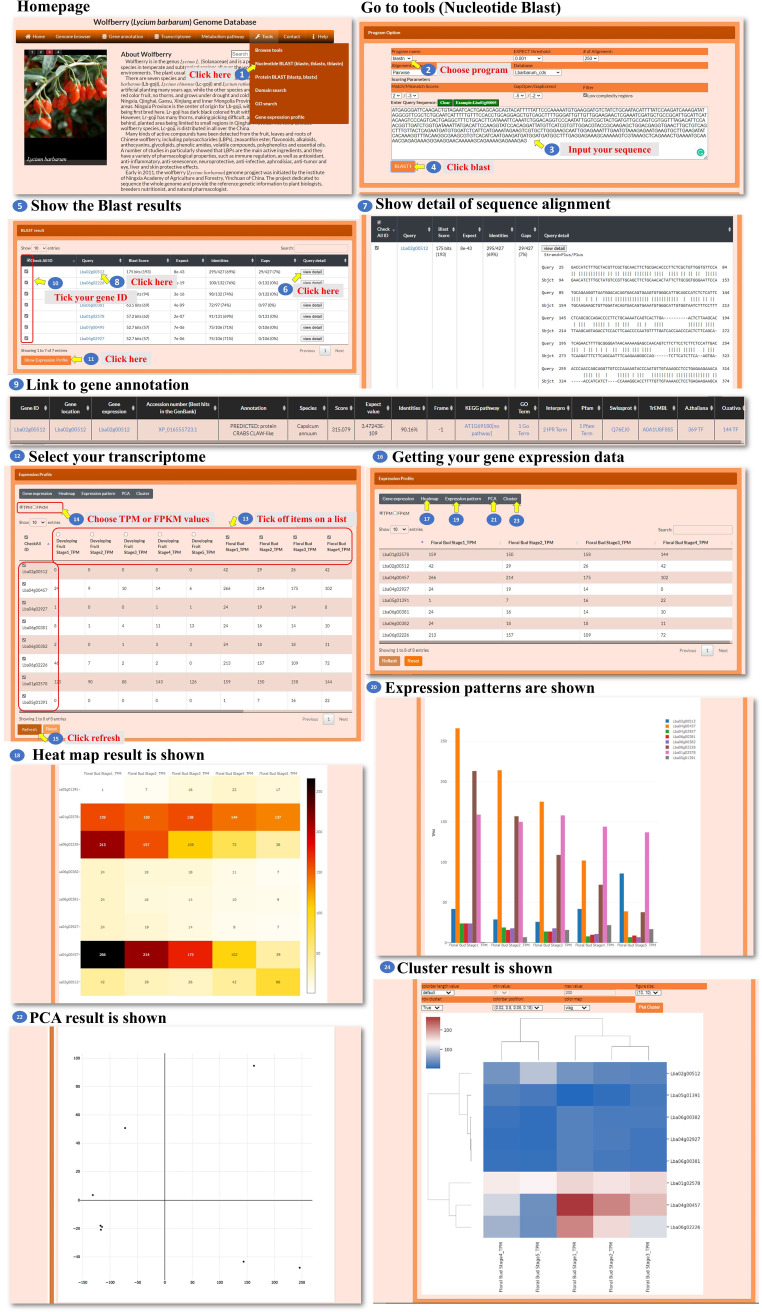
Step-by-step guide for the Tools_Nucleotide BLAST page.

**Figure 9:**
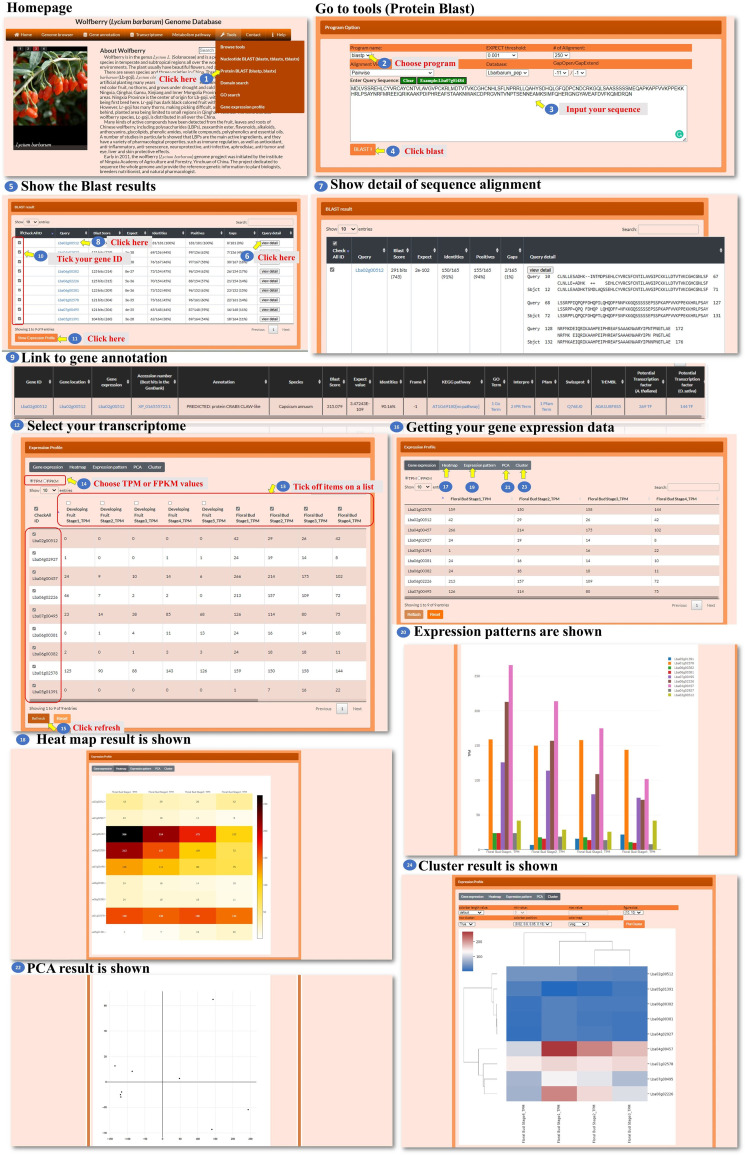
Step-by-step guide for the Tools_Protein BLAST page.

In the tools for nucleotide BLAST and protein BLAST, the user can input a nucleotide sequence or a protein sequence to search for sequence fragments of *L. barbarum*. These sequences could be pasted into the designated area within the tools (**Figures 8**, **9**, steps 1 to 4). After performing the BLAST search, a BLAST result page is presented. This page displays detailed information about the sequence alignment, allowing the user to examine how the input sequence aligns with the *L. barbarum* genome or protein sequences (**Figures 8**, **9**, steps 5 to 7). The user can find the gene annotation on the BLAST result page (**Figures 8**, **9**, steps 8 to 9). This information provides insights into the annotated genes associated with the sequence fragments of interest in *L. barbarum*. The BLAST result page also allows the user to select any of the hit sequences to view their expression profiles. The user could find expression data for the selected sequences, which can provide valuable insights into their transcriptional activity or abundance across different conditions (**Figures 8**, **9**, steps 10 to 11). On the expression profile page, the user can select the transcriptomes and genes they want to compare. They can choose metrics such as TPM (transcripts per million) or FPKM (fragments per kilobase of transcript per million mapped reads) to quantify gene expression. Clicking on “refresh” initiates the comparison process (**Figures 8**, **9**, steps 12 to 15). The expression profile page offers various ways to visualize the comparison results, such as a heatmap, bar chart, principal component analysis (PCA), or hierarchical clustergram. The user can choose the preferred visualization method based on their needs and preferences (**Figures 8**, **9**, steps 16 to 24). Overall, the Nucleotide BLAST and Protein BLAST tools allow the user to search for sequence fragments in the *L. barbarum* genome or predicted proteome. The tools provide detailed sequence alignment information, gene annotation, and expression profiles for the hit sequences. Additionally, the expression profile page offers multiple visualization options to facilitate the analysis and interpretation of the comparison results.

**Figure 10:**
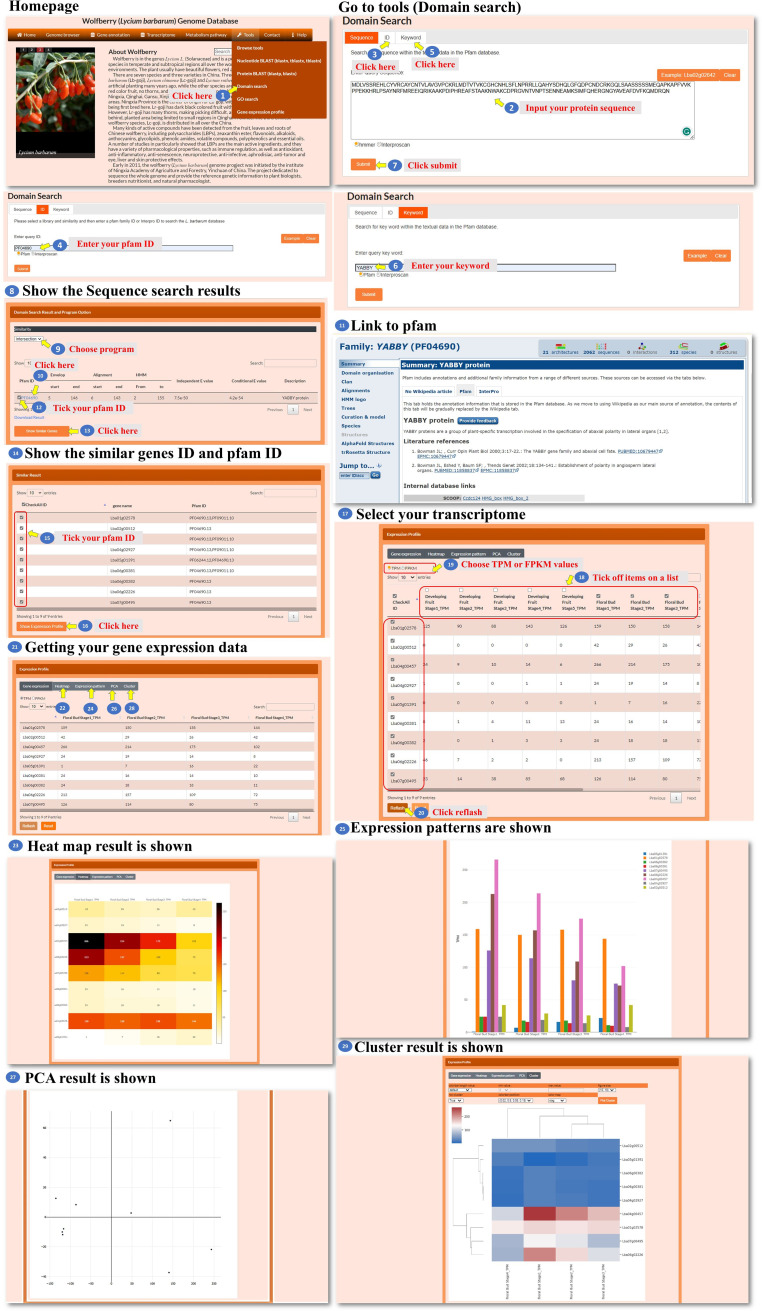
Step-by-step guide for the Tools_Domain search page.

The Domain search tool allows the user to search for sequences or keywords associated with specific Pfam or Interpro IDs. The user can enter a query by sequence, gene ID, or keyword (**Figure 10**, steps 1 to 6). The tool takes the user’s input and performs a search based on the specified sequence, gene ID, or keyword and processes the query and identifies relevant Pfam or Interpro IDs associated with the input. Then, the tool presents the search results to the user, displaying the identified Pfam or Interpro IDs that match the query (**Figure 10**, step 7). The search result webpage allows the user to choose one of three methods, Jaccard, intersection, or union (**Figure 10**, step 8), which is performed by similarity comparison and then determines how the domains are screened for inclusion in the query. Using the selected method, the tool filters the Pfam or Interpro domains and presents the refined list to the user (**Figure 10**, step 9). The user can click on a specific Pfam or Interpro ID to access the corresponding domain’s characteristics (**Figure 10**, steps 10 to 11). From the domain characteristic page, the user has the option to select several Pfam or Interpro IDs to search for similar genes (**Figure 10**, steps 12 to 14). The user can further select a gene ID from the search results to access the transcriptomic data associated with the genes (**Figure 10**, steps 15 to 20). In the new page, the user could choose a heatmap, bar chart, PCA, or hierarchical clustergram to visualize the expression patterns of the selected genes (**Figure 10**, steps 21 to 29). This comprehensive tool enables the user to search for domains, explore domain characteristics, find similar genes, and analyze the transcriptomic data of selected genes using various visualization methods.

**Figure 11:**
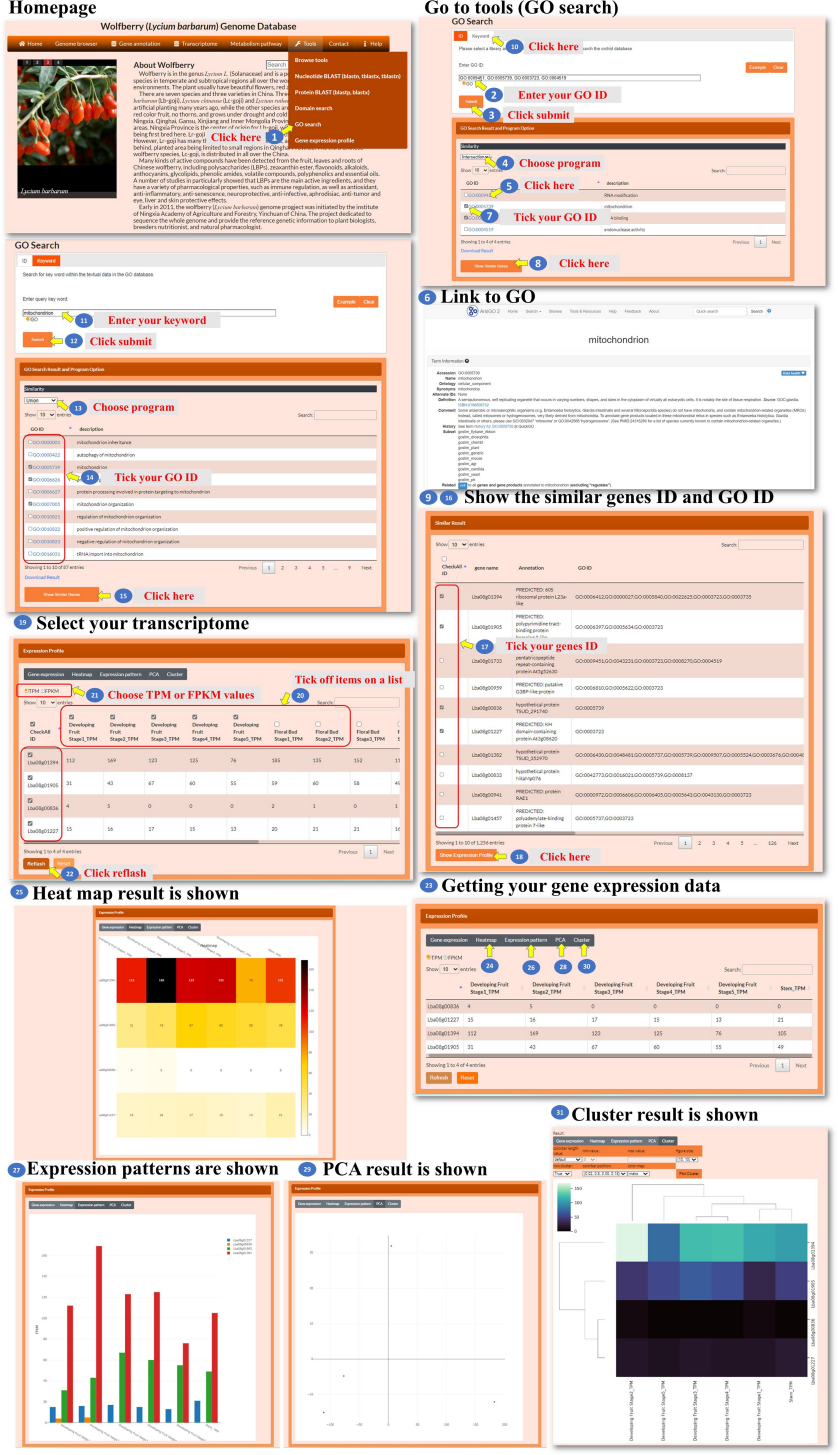
Step-by-step guide for the Tools_GO search page.

For the gene ontology (GO) search, the user enters the GO ID in the search box to query the gene ontology (**Figure 11**, steps 1 to 2). The tool processes the query and retrieves the relevant search results. The search result webpage displays the retrieved results (**Figure 11**, step 3). The user could select one of three methods, Jaccard, intersection, or union, to filter the GO ID results accordingly (**Figure 11**, step 4). Then, the user could click the GO ID to visit the interested GO characteristic to explore GO annotation and gather information about the selected GO ID (**Figure 11**, steps 5 to 6). The user has the option to select the GO ID for searching similar genes. The tool performs a search based on the selected GO ID and retrieves genes with similar annotations (**Figure 11**, steps 7 to 9). The user could also use keywords to query the GO content (**Figure 11**, steps 10 to 12). The user can select one of the similarity comparison methods to filter the GO content results (**Figure 11**, step 13). The user can further select several GO IDs for searching similar genes (**Figure 11**, steps 14 to 16). After obtaining the list of similar genes, the user can further select several gene IDs for analysis (**Figure 11**, steps 17 to 20). The tool retrieves the transcriptomic data for the selected genes. In the new page, the user has the option to choose from different visualization methods such as a heatmap, bar chart, PCA plot, or hierarchical clustergram (**Figure 11**, steps 21 to 29). This GO search tool allows the user to query by GO ID or keyword, explore GO characteristics and annotations, find similar genes based on GO annotations, and analyze the transcriptomic data of selected genes using various visualization methods.

**Figure 12:**
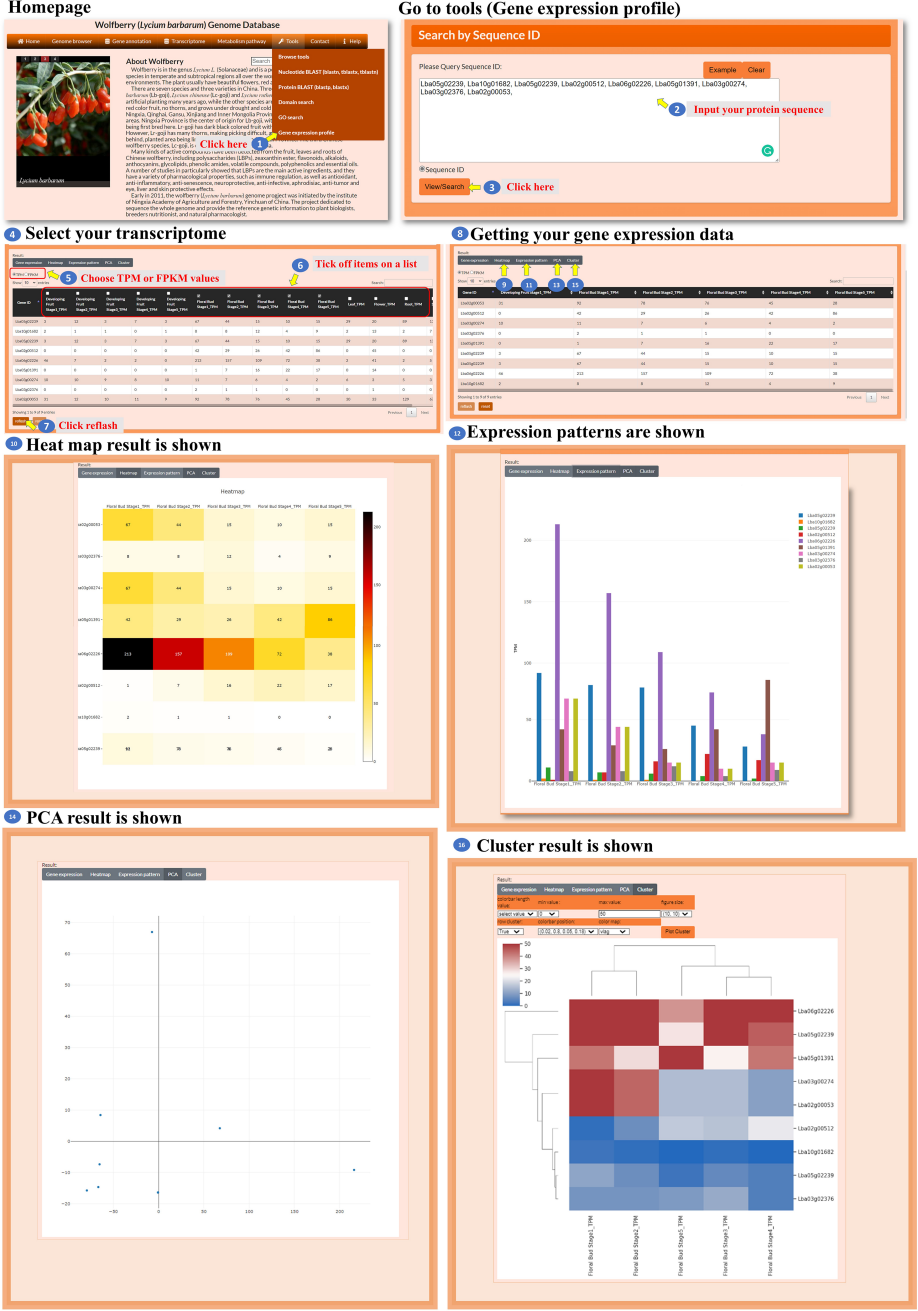
Step-by-step guide for the Tools_Gene expression profile.

The database also provides a tool for analyzing the expression profiles of the user’s genes of interest (**Figure 12**, step 1). The user enters specific gene IDs in the provided box to retrieve the transcriptomic data for those genes (**Figure 12**, steps 2 to 3). The user has the option to select either TPM or FPKM as the expression evaluation method (**Figure 12**, step 5) and choose spatial and temporal expression items for analysis (**Figure 12**, steps 6 to 7). After acquiring the expression data, the user can select a visualization method, such as a heatmap, bar chart, PCA, or hierarchical clustergram, to display the expression profiles (**Figure 12**, steps 8 to 16). This tool enables the user to analyze the expression profiles of their genes of interest by inputting specific gene IDs, selecting expression evaluation methods, considering spatial and temporal expression aspects, and visualizing the expression data using different visualization options.

### A case study

**Figure 13:**
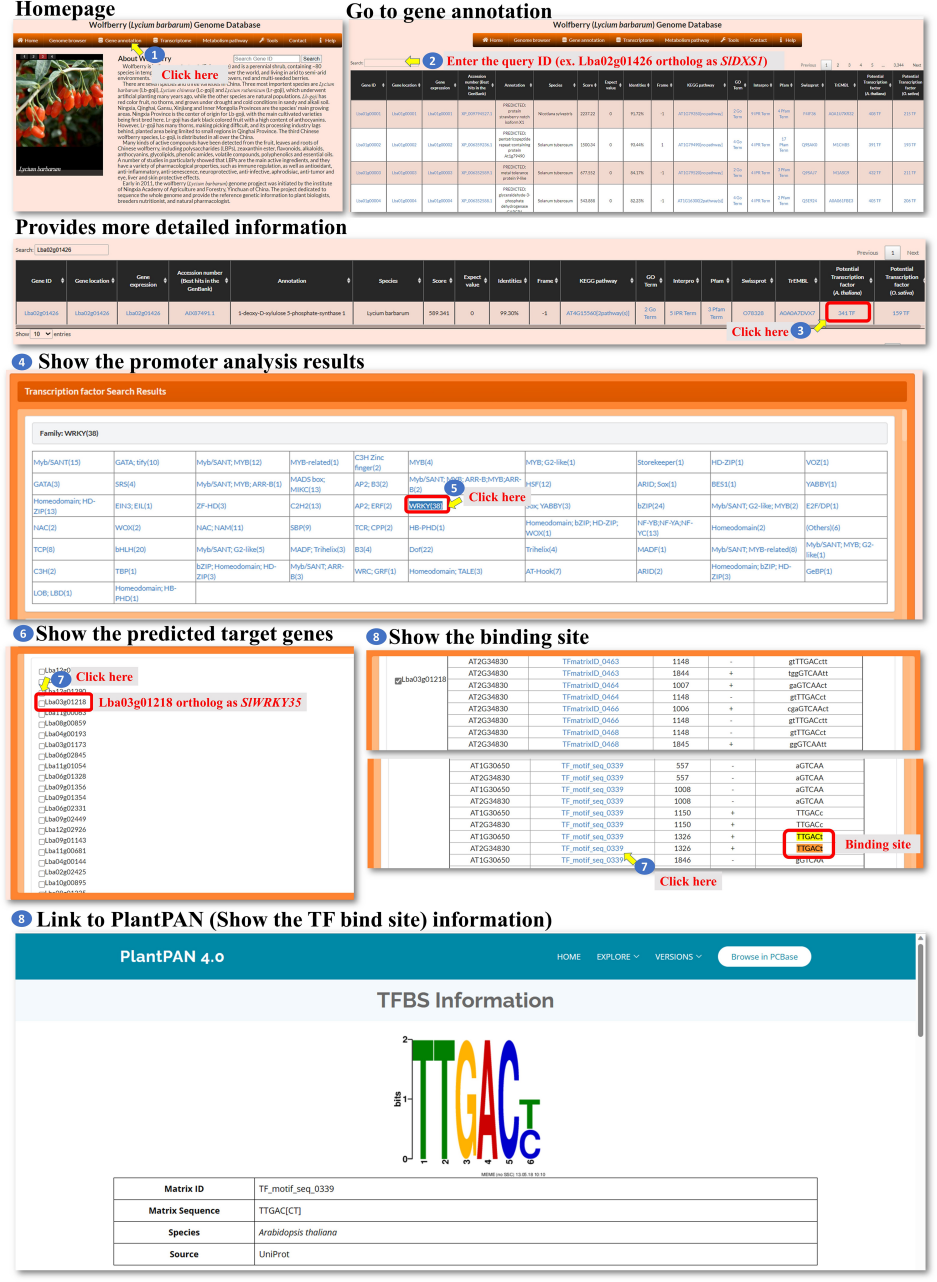
A Case study showing the analysis steps of WRKY transcri ption factor regulating the carotenoids biosynthesis.

*L. barbarum* has gained popularity as a “superfood” due to its potential health benefits. One of the key components that contributes to their nutritional value is their carotenoid content. Carotenoids participate in a wide range of plant physiological processes, including light harvesting, photosynthesis, plant growth, and responses to biotic and abiotic stresses (Xiong et al., 2019). We are interested in the regulation of carotenoid biosynthesis in *L. barbarum*, and we investigated the possible genes involved in *L. barbarum* carotenoid production. It has been shown that carotenoids are synthesized through the plastid methyl-erythritol phosphate (MEP) pathway and positively regulated by the WRKY transcription factor in tomato fruit (Yuan et al., 2022). Because *L. barbarum* and tomato are classified in the same Solanaceae family, it is reasonable to predict that carotenoids accumulated in *L. barbarum* fruit might be regulated in a similar way. First, we identified the *L. barbarum* orthologous gene (*Lba02g01426*) of the tomato *1-deoxy-D-xylulose 5-phosphate synthase* gene (*SlDXS1*) via BLASTP, the gene encoding the enzyme that produces 1-deoxy-D-xylulose 5-phosphate from glyceraldehyde‐3‐phosphate (GAP) and pyruvate in the MEP pathway for isoprenoid biosynthesis. Then, we went to the gene annotation page and entered Lba02g01426 to query its detailed annotation (**Figure 13**, steps 1 to 2). Three hundred forty-one transcription factors that putatively regulate *Lba02g01426* expression could be seen (**Figure 13**, step 3). After clicking the 341 TF marked in red, we could then see the thirty-eight genes encoding WRKY transcription factors that might bind to the promoter of *Lba02g01426* (**Figure 13**, steps 4 to 5). WRKY transcription factors have been reported to control carotenoid accumulation in several dicot plants, such as sweet osmanthus, citrus, and tomato (Han et al., 2016; Jiang et al., 2019; Duan et al., 2022; Yuan et al., 2022). Click WRKY (38), and we could see the predicted WRKY list (**Figure 13**, step 6). Because *Lba03g01218* is the orthologue of tomato *SlWRKY35*, we could select this gene and find its binding site (**Figure 13**, steps 7 and 8). The binding site shown here is highly similar to that of tomato SlWRKY35 (Yuan et al., 2022). Overall, our investigation provides valuable insights into the potential regulation of carotenoid biosynthesis in *L. barbarum* and highlights the involvement of WRKY transcription factors as potential regulators of this process.

## Conclusion

Although several genomic databases related to Solanaceae have been reported, such as the Solanaceae Genomic Network (Fernandez-Pozo et al., 2014), PoMaMo (Meyer et al., 2005), PepperHub (Liu et al., 2017), and *Nicotiana attenuata* Data Hub (Brockmöllere et al., 2017), the genome database of wolfberry is still absent. Thanks to advanced sequencing technology, the *L. barbarum* genome has been successfully sequenced (Cao et al., 2021). Our WGDB provides an integrative public platform for sharing and analyzing research data and easy-to-use web interfaces. The availability of WGDB will facilitate various studies related to wolfberry, such as understanding its genetics, identifying genes responsible for specific traits or functions, and exploring potential applications in agriculture, biotechnology, and pharmacology. Researchers interested in wolfberry biology will now have easy access to data and a user-friendly interface to explore, analyze, and gain insights into the molecular aspects of this plant.

## Data availability statement

The raw data and whole genome−assembled scaffold sequences for the Licium barbarum (PRJNA640228), (https://www.ncbi.nlm.nih.gov/bioproject/?term=PRJNA640228) were downloaded from the National Center for Biotechnology Information (NCBI) database. The transcriptome data were downloaded from PRJNA788208, (https://www.ncbi.nlm.nih.gov/bioproject/?term=PRJNA788208).

## Author contributions

W-CT: Conceptualization, Formal analysis, Funding acquisition, Project administration, Resources, Supervision, Writing – original draft, Writing – review & editing. Y-LC: Data curation, Funding acquisition, Project administration, Resources, Supervision, Validation, Writing – review & editing. Y-YC: Data curation, Formal analysis, Investigation, Methodology, Validation, Writing – review & editing. Y-LL: Data curation, Formal analysis, Investigation, Methodology, Project administration, Resources, Validation, Writing – review & editing. C-IL: Data curation, Investigation, Methodology, Software, Validation, Writing – review & editing. S-TL: Methodology, Validation, Writing – review & editing. B-RL: Data curation, Methodology, Software, Visualization, Writing – review & editing. C-LH: Data curation, Methodology, Software, Visualization, Writing – review & editing. Y-YH: Data curation, Formal analysis, Investigation, Validation, Writing – review & editing. Y-FF: Data curation, Formal analysis, Methodology, Validation, Writing – review & editing. QL: Data curation, Methodology, Validation, Writing – review & editing. J-HZ: Data curation, Investigation, Validation, Writing – review & editing. YY: Data curation, Formal analysis, Validation, Writing – review & editing. WA: Data curation, Formal analysis, Investigation, Writing – review & editing. Z-GS: Data curation, Investigation, Validation, Writing – review & editing. C-NC: Data curation, Methodology, Validation, Writing – review & editing. W-CC: Formal analysis, Investigation, Methodology, Validation, Writing – review & editing. C-LH: Data curation, Formal analysis, Investigation, Writing – review & editing. W-HC: Investigation, Writing – review & editing. Z-JL: Conceptualization, Funding acquisition, Investigation, Resources, Supervision, Validation, Writing – review & editing. W-SW: Conceptualization, Formal analysis, Funding acquisition, Methodology, Software, Supervision, Validation, Visualization, Writing – original draft, Writing – review & editing.
